# The therapeutic potential of compounds from natural products for alleviating depression by targeting P2X7 receptor-mediated proinflammatory signaling pathways

**DOI:** 10.3389/fimmu.2026.1814913

**Published:** 2026-04-02

**Authors:** Xin-Yang Bai, Gui-Lin Wang, Yue Zhang, Qian Cheng, Hui-Ning Feng, Linyu Wei, Hai-Yan Sheng, Yaling Yin, Sebastien Roger, Lin-Hua Jiang, Huifang Hou

**Affiliations:** 1 Department of Physiology and Pathophysiology and Sino-UK Joint Laboratory of Brain Function and Injury of Henan Province, Henan Medical University, Xinxiang, China; 2 Henan Key Laboratory of Neurorestoratology and Protein Modification, Henan Medical University, Xinxiang, China; 3 INSERM U1327 ISCHEMIA ‘Membrane Signalling and Inflammation in Reperfusion Injuries’, Faculty of Medicine, University of Tours, Tours, France; 4 School of Biomedical Sciences, Faculty of Biological Sciences, University of Leeds, Leeds, United Kingdom

**Keywords:** antidepressant effects, depression, natural products, neuroinflammation, P2X7 receptor

## Abstract

Depression is a prevalent neuropsychiatric disorder with high incidence and causing severe disability, representing a clinically unmet challenge and thus demanding more effective therapeutics. Neuroinflammation in the central nervous system (CNS) is a pathological feature of depression and, with increasing recognition, it is also a critical depression-driving mechanism. In the CNS, the P2X7 receptor for extracellular ATP is expressed in microglia and astrocytes, and acts as a key mediator of neuroinflammation. Besides medicinal chemistry efforts in developing novel CNS-penetrable P2X7 antagonists, there is an increasing interest in exploring natural products as medications for CNS conditions including depression. In this mini-review, we discuss the recent progress in examining the therapeutic potential and mechanisms of compounds from natural products, using rodent models of depression, and revealing P2X7-mediated proinflammatory signaling pathways as an important target for their antidepressant actions.

## Introduction

Major depressive disorder or depression is a common neuropsychiatric disorder, with an estimated 4% of the world population or 5.7% of adults suffering depression according to the World Health Organization (1), and manifested often in comorbidity with other conditions (2–4). Depression remains a significant burden to public health worldwide (5, 6). The currently available medications acting on the monoamine neurotransmission systems are grieved with limited efficacy, slow onset of action and considerable side effects, demanding more effective therapeutics. Decades of researches indicate multifaceted etiology of depression and complex underlying mechanisms (7). Preclinical and clinical studies have gathered persuasive evidence to support neuroinflammation in the CNS as a critical pathogenic mechanism of depression (8–10). In the CNS, microglia are the major immunocompetent cells and play a key role in mediating neuroinflammation that induces aberrant synaptic modeling and neuronal death, which are strongly implicated in depression pathogenesis, and as such depression has been even portrayed as a microglial disorder (9).

P2X receptors are a family of ATP-gated Ca^2+^-permeable ion channels, and P2X7 is an unusual member for its activation requiring high concentrations of ATP and its role in mediating cell death (11). P2X7 is expressed in cells of the immune system and, upon activation, can stimulate multiple pro-inflammatory signaling pathways, e.g., the NOD-like receptor family pyrin domain-containing 3 (NLRP3)/caspase-1/gasdermin D (GSDMD) pathway, generate proinflammatory mediators, such as interleukin (IL)-1β, IL-6, tumor necrosis factor (TNF)-α and reactive oxygen species (ROS) and, furthermore, induce proinflammatory pyroptosis, therefore acting as a key player in immunity and inflammatory diseases (12). In the CNS, P2X7 is highly expressed in microglia and also present in astrocytes, and mediates neuroinflammation associated with multiple traumatic damage and neurogenerative diseases (13–18). Increasing evidence indicates excessive release of ATP and subsequent P2X7 activation drive neuroinflammation in psychiatric diseases, including depression, and P2X7 becomes an attractive target for developing antidepressant therapeutics (8, 15–19). To explore the therapeutic potential of P2X7, tremendous medicinal chemistry efforts have been devoted, leading to development of a repertoire of novel compounds as P2X7 receptor antagonists, and many of them exhibit desirable CNS penetration, prompting the interest in them as medications for depression and other CNS conditions (8, 14, 20–22). Meanwhile, increasing attentions have been drawn to natural products and compounds with anti-inflammatory, antioxidant and neuroprotective activities, from herbal plants and traditional Chinese medicines (TCM), for their antidepressant potential.

In this mini-review, we discuss recent studies that have revealed a number of compounds from natural products that exhibit a potent antidepressant effect via modulating P2X7-mediated proinflammatory signaling pathways to mitigate neuroinflammation. Such findings highlight herb plants and TCM as a promising additional avenue for developing P2X7 antagonists to treat depression and other neuroinflammation-driven CNS conditions.

## Antidepressant actions of compounds from natural products via P2X7-mediated neuroinflammation

**Figure 1:**
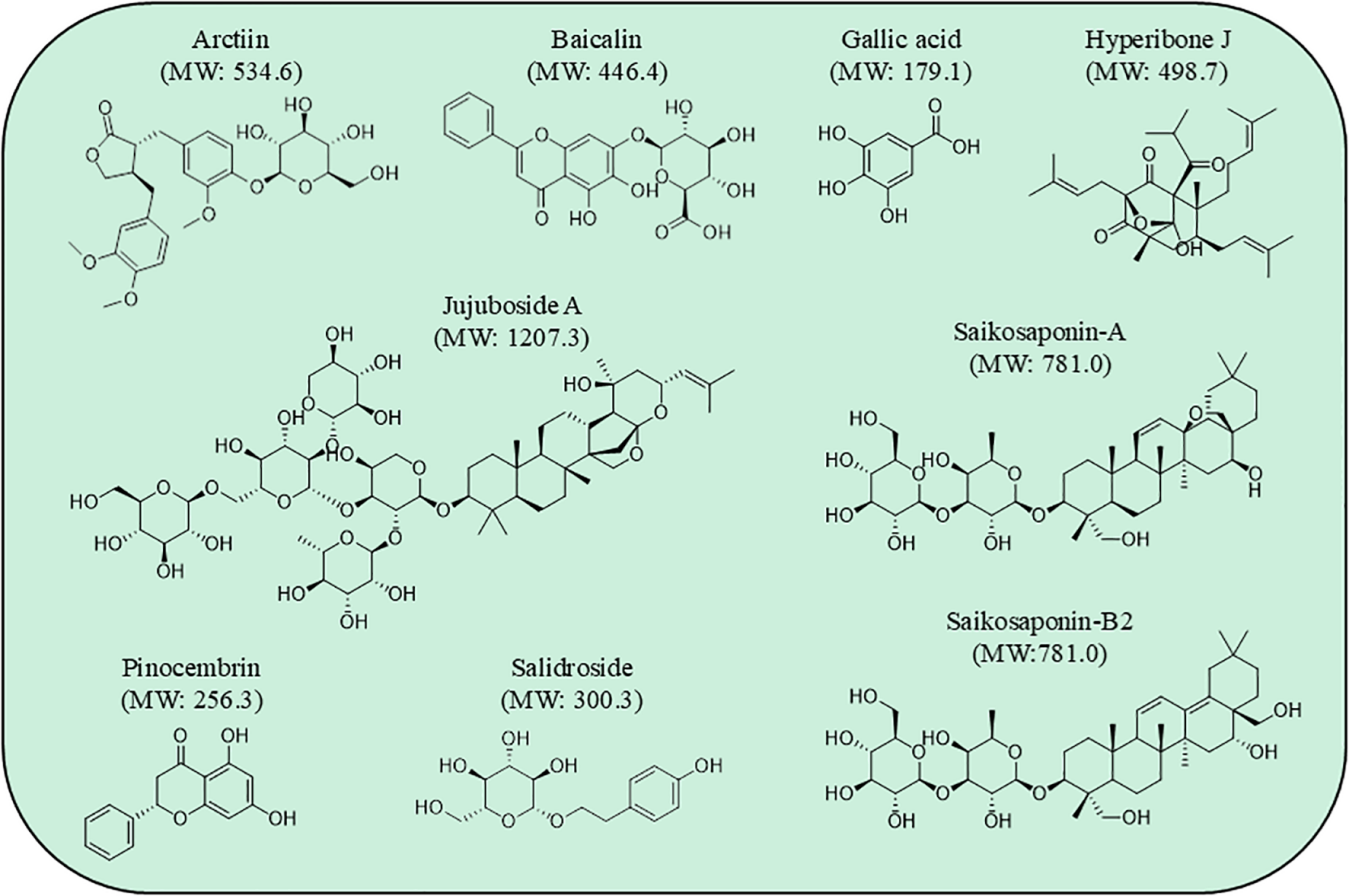
Chemical structure and molecular weight (MW) of the antidepressant compounds from natural products modulating P2X7-mediated proinflammatory pathways.

*Arctiin* or arctigenin-4-glucoside (**Figure 1**) is one of the major bioactive ingredients in Fructus arctii, the dried ripe fruits of Arctium lappa L, a widely-used medicinal herb in Asia (23). Arctiin exhibits an antidepressant activity, and the mechanisms of actions have been examined, in mouse model of depression induced by chronic unpredictable mild stress (CUMS) (21, 24). Oral administration of arctiin, while causing no effect on the spontaneous locomotor activity assessed in the open field test (OFT), reduced depressive-like behaviors by shortening the immobility time in both tail suspension test (TST) and forced swimming test (FST). Arctiin also alleviated anhedonia, one of the key symptoms of depression, as shown in the sucrose preference test (SPT). CUMS upregulated the expression of microglial marker protein, ionized calcium-binding adapter molecule 1, and enhanced the levels of proinflammatory mediators, including high mobility group box 1 (HMGB1), IL-1β, TNF-α and inducible nitric oxide (NO) synthetase (iNOS), in the prefrontal cortex (PFC) and also the levels of IL-1β, TNF-α and NO in the serum. CUMS also caused neuronal loss in the PFC, and reduced the activity of indoleamine 2,3-dioxygenase, the expression of tyrosine hydroxylase and the levels of 5-hydroxytryptamine (5-HT) and dopamine in the PFC. Such CUMS-induced neuroinflammation was alleviated by treatment with arctiin. Arctiin has been proposed in a recent study to alleviate depressive-like behaviors by binding to P2X7 to inhibit the P2X7/NLRP3/caspase-1 pathway (21). Of notice, arctiin was previously hypothesized to bind to the toll-like receptor 4 (TLR4) in microglia to prevent activation of the HMGB1/TLR4 and TNF-α/TNF receptor-1 pathways, which are known to stimulate the nuclear factor-kappa B (NF-κB) pathway, and thereby reduce the expression and generation of pro-inflammatory mediators (24).

*Baicalin* or 5,6-dihydroxy-4-oxo-2-phenyl-4H-chromen-7-yl beta-D-glucopyranosiduronic acid (**Figure 1**) is a polyphenol compound isolated from the dry root of *Scutellaria baicalensis* Georgi, also known as Chinese skullcap and used as a medicinal plant for thousands of years in China. Epilepsy is comorbid with anxiety and depression. As shown in rat model of epilepsy induced by pentylenetetrazol kindling, intragastric administration of baicalin has recently been shown to ameliorate anxiety-like and depressive-like behaviors in the OFT and FST, as well as the severity of seizures (25). Brain-derived neurotrophic factor (BDNF) and its cognate tyrosine kinase receptor B (TrkB) are known to drive gene expression and thereby modulate synaptic plasticity and neurogenesis, and deficient BDNF/TrkB signaling has been implicated in depression pathogenesis (26, 27). Baicalin prevented pentylenetetrazol-induced decrease in the number of neurons and the BDNF levels and promoted neurogenesis in the hippocampus. In addition, baicalin suppressed pentylenetetrazol-induced upregulation in the expression of P2X7, NLRP3 and IL-1β in microglia, supporting the notion that baicalin inhibits the P2X7/NLRP3 pathway for its ability of alleviating epilepsy and comorbid anxiety and depression (25). In CUMS-exposed rats, intragastric administration of baicalin also improved the spontaneous locomotive activity in the OFT and mitigated depressive-like behaviors and anhedonia in the FST and SPT via reducing neuroinflammation (28). Baicalin has been also proposed to exert an antidepressant effect via multiple distinctive mechanisms, including inhibition of α-amino-3-hydroxy-5-methyl-4-isoxazolepropionic acid receptor-mediated excitotoxicity (29) or stimulation of glucocorticoid receptor-mediated neurogenesis (23).

*Gallic acid* (GA) or 3,4,5-trihydroxybenzoic acid (**Figure 1**) is a polyphenolic compound present in plants, such as green tea and grapes (30). Its antidepressant mechanism has been investigated in rat model of depression induced by neonatal colorectal distension (CRD) (31). Oral administration of GA alleviated depressive-like behaviors in the FST and anhedonia in the SPT, without affecting the spontaneous locomotion in the OFT. GA inhibited CRD-induced upregulation of the P2X7 expression and activation of astrocytes and, moreover, reduced the levels of IL-1β and TNF-α and increased the levels of anti-inflammatory cytokine IL-10 and BDNF in the hippocampus (31). As alluded above, pain and depression are comorbid, and neuroinflammation plays a critical role in such comorbidity. In rat pain and depression comorbidity model induced by chronic constrictive injury (CCI) followed by CUMS, oral administration of GA reversed the pain hypersensitivity and alleviated depressive-like behaviors and anhedonia in the FST and SPT (30). GA restored the levels of glutathione and glutathione peroxidase 4, reduced the levels of ROS and malondialdehyde (MDA), a byproduct of lipid peroxidation, and tissue iron content, and ameliorated mitochondrial damage in the spinal cord (30). The antidepressant actions of GA have been attributed to inhibition of P2X7 and, as a result, suppress the activity of TNF-α−converting enzyme, which generate TNF-α, and inhibit the NF-κB/signal transducer and activator of transcription 3 (STAT3) pathway, thereby reducing ROS generation, lipid peroxidation and iron deposition that drive ferroptosis of microglia in the spinal cord (30). In addition, GA was proposed to inhibit the extracellular signal-regulated kinase (ERK) pathway, reduce the levels of IL-1β and TNF-α and increase the levels of IL-10 and BDNF in the hippocampus (31).

*Hyperibone J* (**Figure 1**) is a principal polyprenylated acylphloroglucinol component with a complex adamantane-like core structure, isolated from the flowers of Hypericum bellum (32). Its antidepressant action has been recently examined in CUMS-exposed mice or in mice exposed to lipopolysaccharide (LPS), a potent inducer of neuroinflammation (32). Oral administration of Hyperibone J alleviated depressive-like behaviors in the TST and FST and anhedonia in the SPT and also improved exploratory and anxiety-related behaviors in the OFT. Such antidepressant effects were blunted by inhibiting P2X7. CUMS or LPS induced microglial activation, and increased the levels of IL-1β, IL-6 and TNF-α and neuronal damage in the hippocampus, which were inhibited by Hyperibone J. Hyperibone J has been proposed to bind to adenosine kinase to promote its degradation, and prevent excessive production and release of ATP and subsequent P2X7 activation as well as downregulation of the TLR4 expression, thereby dampening the TLR4/NF-κB and NLRP3/caspase-1 pathways and together reducing generation of pro-inflammatory mediators (32).

*Jujuboside A (JuA)* is a triterpenoid (**Figure 1**) and a major bioactive saponin isolated from Ziziphus jujuba seeds. JuA has been assessed recently for its effects on depression comorbid with visceral pain in rats (33). Oral treatment with JuA improved pain-related CRD-induced depressive-like behaviors and anhedonia in the FST and SPT, similarly to intrathecal injection of shRNA to reduce P2X7 expression or MCC950 to inhibit NLRP3 activation. JuA prevented CRD-induced upregulation of the P2X7R expression in astrocytes and reduction in the BDNF level in the spinal cord and hippocampus. JuA also abolished CRD-induced upregulation in the expression of NLRP3, caspase-1 and GSDMD in the spinal cord and hippocampus and, furthermore, caspase-3 in the hippocampus. JuA prevented CRD-induced neuronal death in the hippocampus. As demonstrated in cultured astrocytes, JuA inhibited astrocyte death and generation of IL-1β and TNF-α, induced by combined treatment with substance P and corticosterone that mimics the pathological state of visceral pain with comorbid depression. In hippocampal neurons cultured in the medium conditioned by substance P/corticosterone-treated astrocytes, the BDNF level was reduced and apoptosis induced, both of which were alleviated by treating astrocytes with JuA. These results have been interpreted to indicate that JuA targets P2X7 in astrocytes to inhibit the NLRP3/caspase-1/GSDMD pathway, thereby preventing astrocytes from generation of proinflammatory cytokines and pyroptosis (33).

*Pinocembrin* or 5,7-dihydroxyflavanone (**Figure 1**) is a natural flavonoid compound found in propolis, honey and plants of the Pinus and Eucalyptus genera (13). Its antidepressant mechanism has been recently evaluated in CUMS-exposed mice. Oral administration of pinocembrin alleviated depressive-like behaviors in the FST and anhedonia in the SPT and, additionally, improved the exploratory behaviors without affecting the spontaneous locomotor activity in the OFT (13, 34). CUMS induced oxidative stress in the hippocampus, manifested by increased levels of ROS and MDA, and decreased activity of superoxide dismutase (SOD). Furthermore, CUMS induced caspase-3 activation and neuronal apoptosis (13), and downregulated the expression of synaptic proteins, postsynaptic protein-95 and synapsin (34). These CUMS-induced pathologies were reversed by treatment with pinocembrin (13, 34). In CUMS-exposed mice, intraperitoneal administration of pinocembrin also mitigated depressive-like behaviors in the TST and anhedonia in the SPT (35). CUMS induced microglial activation, and promoted polarization towards the inflammatory phenotype, indicated by upregulated expression levels of CD68 and CD16 and generation of IL-1β, IL-6 and TNF-α in the hippocampus, which were abolished by pinocembrin (35). The antidepressant action of pinocembrin has been proposed to prevent CUMS-induced upregulation in the expression of P2X7R and TLR4 in the hippocampus and thereby suppress the NLRP3/caspase-1 and TLR4/NF-κB pathways (34, 35). Of notice, pinocembrin was also proposed to activate the nuclear factor erythroid 2-related factor 2/heme oxygenase-1 antioxidant pathway and inhibit the NF-κB pathway (13). Pinocembrin has been further reported to inhibit P2X4-mediated astrocyte pyroptosis in the hippocampus to alleviate chronic pain and comorbid depressive-like behaviors in rats (36).

*Saikosaponins*, a class of triterpenoid saponins that constitute the principal bioactive ingredients of Bupleurum specie (37, 38). The antidepressant mechanisms of saikosaponins have been investigated in multiple models. In LPS-exposed mice, oral treatment with saikosaponins alleviated depressive-like behaviors in the FST and TST and anhedonia in the SPT (38). In CUMS-exposed mice, oral treatment with monomer components, including saikosaponin-A and saikosaponin-B2 (**Figure 1**), improved depressive-like behaviors and anhedonia in the FST and SPT, without affecting the spontaneous locomotor activity in the OFT (39, 40). Saikosaponins attenuated microglial activation and generation of IL-1β, IL-6, and TNF-α in the hippocampus (38, 39, 41), protected neuronal damage and upregulated the BDNF expression (40, 41). Saikosaponins also normalized the activity of the hypothalamic-pituitary-adrenal axis, which plays a vital role in mediating depression, and elevated the dopamine level in hippocampus (40) and the levels of 5-HT, norepinephrine and dopamine in the striatum (41). Saikosaponin -B2 was shown to suppress the TLR4/NF-κB pathway and saikosaponin -A activate the neuroplasticity-related pathways, including the cAMP response element-binding protein (CREB)/BDNF and ERK pathways (39, 40). Therefore, inhibition of multiple pathways underpins the antidepressant action of saikosaponins. A recent study pinpoints an interaction of saikosaponins with P2X7 as a key mechanism of action, in which saikosaponins reduce the P2X7 expression and thereby the P2X7/NLRP3/caspase-1/GSDMD pathway in the brain (38).

*Salidroside* or 2-(4-hydroxyphenyl) ethyl β-D-glucopyranoside (**Figure 1**) is the main bioactive phenylpropane glycoside component of Rhodiola rosea L, a traditional medicinal herb (42). Its antidepressant mechanism has been examined using mouse models of depression induced by corticosterone, LPS or chronic unpredictable stress (CUS) (43–45). In corticosterone or CUS-exposed mice, oral administration of salidroside, while having no effect on the spontaneous locomotor activity in the OFT, produced an antidepressant effect in the FST and alleviated anhedonia in the SPT. In LPS-exposed mice, oral administration of salidroside improved anhedonia in the SPT. Corticosterone or CUS caused neuronal apoptosis in the hippocampus, leading to a reduction in the number of neurons and also a reduction in the BDNF level in the hippocampus (43, 44). Corticosterone or CUS also induced microglial activation, indicated by increases in the number of CD68-positive cells and the levels of IL-1β and TNF-α in the hippocampus (44). Such neuroinflammation was reversed by treatment with salidroside (43, 44). Salidroside has been hypothesized to inhibit P2X7 to suppress the NF-κB and NLRP3/caspase-1/GSDMD pathways that drive generation of proinflammatory cytokines and pyroptosis in microglia (44, 45).

*Morinda officinalis oligosaccharides* (MOOs) contain diverse oligosaccharides and are extracted from Morinda officinalis roots and, have been prescribed as a TCM to treat depression (46). Post-stroke depression (PSD) represents an important complication of stroke, predisposing patients to greater disability and mortality (47). In rat model of PSD induced by transient ischemia via middle cerebral artery occlusion (MCAO) followed by CUMS, oral administration of MOOs attenuated depressive-like behaviors in the FST and TST, without affecting the spontaneous locomotor activity in the OFT (48). MOOs have been proposed to suppress the NF-κB pathway to downregulate the NLRP3 expression in microglia and thereby dampen P2X7-dependent activation of the NLRP3/capase-1 pathway (48). In rat model of depression induced by CUS, intragastric administration of MOOs also alleviated depressive-like behaviors in the FST and anhedonia in the SPT (49). CUS reduced the BDNF level, and impaired neurogenesis and synaptic function in the PFC and hippocampus, which were reversed by treatment with MOOs (49). Similarly, in CUMS-induced rats, oral administration of MOOs mitigated anhedonia in the SPT (50). The antidepressant action of MOOs was proposed to increase the expression of tryptophan hydroxylase that accelerates 5-HTP production from tryptophan and decrease the activity of 5-HTP decarboxylase that reduces 5-HT generation, leading to accumulation of 5-HTP in the gut microbiota, which reaches the brain to elevate 5-HT generation and relive depression (50).

## Conclusions and perspectives

P2X7, alongside the NLRP3 inflammasome and proinflammatory cytokines (10, 51), as a key mediator of neuroinflammation has gained continuous attentions as a therapeutic target for multiple CNS conditions including depression. As discussed above and briefly summarized in **Table 1**, recent studies using rodent models have revealed that a structural diversity of compounds from natural products can target neuroinflammation via multiple P2X7-mediated proinflammatory pathways to generate an antidepressant effect. It should be pointed out that most of the compounds remain to be tested as an antidepressant in clinical settings and, as mentioned above, saikosaponins (and MOOs) are long used in TCM to manage depression (37, 38, 47). These findings provide consistent evidence to support the inflammation hypothesis of depression in general and the critical role of P2X7 in mediating depression in particular and open a new avenue to develop P2X7 antagonists as antidepressant medications. An increasing number of compounds from natural products have recently been reported that modulate P2X7-mediated mechanisms, e.g., ginseng and platycodon grandiflorum (52), polydatin (53), vitexin (54), embelin (55), polycyclic aromatic naphthodianthrone (56) and cinobufagin (57) and, it is therefore interesting to examine their antidepressant potential. With continuous efforts devoted to defining the mechanisms of actions of natural products and improving their pharmacokinetics and pharmacodynamics including CNS penetration, natural products and compounds from them can provide benefits to patients suffering from depression and comorbid conditions.

**Table 1:** Summary of compounds from natural products exhibiting an antidepressant effect via P2X7-mediated proinflammatory pathways in rodent models.

Compounds (Main sources)	PubChem CID	Model (& dosing)	Antidepressant effects	Proposed mechanisms	Ref
Arctiin (Fructus arctii)	100528	CUMS-induced mouse model of depression (50, 100 mg/kg; oral)	Inhibiting CUMS-induced increase in immobility in TST and FST and decrease in sucrose preference in SPT.	Binding to P2X7 to inhibit activation of the NLRP3/capase-1 pathway in microglia.	(21)
		MCAO&CUMS-induced rat model of PSD in rats (100 mg/kg; oral)	Inhibiting MCAO&CUMS-induced increase in immobility in FST and TST.	Inhibiting NF-κB to downregulate NLRP3 and activation of the NLRP3/caspase-1 pathway in microglia.	(48)
Baicalin (Scutellaria baicalensis Georgi)	64982	PTZ-induced epilepsy and comorbid with anxiety and depression in rats (100 mg/kg; intragastric).	Ameliorating anxiety-like and depressive-like behaviors in the OFT and FST.	Inhibiting upregulation of P2X7 and NLRP3 in microglia to inhibit the P2X7/NLRP3 pathway.	(25)
Gallic acid (Green tea and grapes)	370	Neonatal CRD-induced rat model of depression (20 mg/kg; oral)	Inhibiting CRD-induced increase in immobility in FST and decrease in sucrose consumption in SPT.	Inhibiting P2X7 upregulation and activation of the ERK pathway in astrocytes.	(31)
		CCI&CUMS-induced rat model of pain and depression (100 mg/kg; oral)	Inhibiting CCI&CUMS-induced increase in immobility in FST and decrease in sucrose preference in SPT.	Binding to P2X7 to suppress activation of the TACE/TNF-α/NF-κB/STAT3 pathway in microglia.	(30)
Hyperibone J (Hypericum bellum)	44575718	CUMS or LPS-induced mouse model of pression (20, 40 mg/kg; oral)	Inhibiting CUMS or LPS-induced increase in immobility in TST and FST and decrease in sucrose preference in SPT and improving exploratory behaviors and anxiety in OFT.	Binding to ADK to promote its degradation to reduce extracellular ATP and activation of P2X7 and the NLRP3/caspase-1 pathway in microglia.	(58)
Jujuboside A (Ziziphus jujuba)	51346169	CRD-induced rat model of pain and depression (10 mg/kg; oral)	Inhibiting CRD-induced increase in immobility in FST and decrease in sucrose consumption in SPT.	Targeting P2X7 to prevent P2X7 activation and the NLRP3/caspase-1/GSDMD pathway in astrocytes	(33)
Pinocembrin (Propolis, honey, Pinus and Eucalyptus plants)	68071	CUMS or LPS-induced mouse model of depression (20 mg/kg; intraperitoneal)	Inhibiting CUMS or LPS-induced increase in immobility in TST and decrease in sucrose preference in SPT.	Inhibiting P2X7 and TLR4 upregulation and activation of the NLRP3/caspase−1 and TLR4/NF-κB pathways in microglia.	(34, 35)
Saikosaponins (Bupleurum specie)		LPS-induced mouse model of depression (120, 250, 400 mg/kg; oral)	Inhibiting LPS-induced increase in immobility in FST and TST and decrease in sucrose preference in SPT.	Inhibiting P2X7 upregulation in microglia and activation of P2X7 and the NLRP3/caspase-1/GSDMD pathway.	(38)
		PTZ-induced rat model of epilepsy and depression (100 mg/kg; oral)	Inhibiting PTZ-induced increase in immobility in FST and improving spontaneous locomotion in OFT.	Inhibiting upregulation of P2X7 and NLRP3 in microglia to reduce activation of the NLRP3/caspase-1 pathway.	(25)
Salidroside (Rhodiola rosea L)	159278	Corticosterone or LPS-induced mouse model of depression (20, 40 mg/kg; oral)	Inhibiting corticosterone- or LPS-induced increase in immobility in FST and decrease in sucrose preference in SPT.	Binding to P2X7 to suppress activation of the NF-κB and NLRP3/caspase-1/GSDMD pathways in microglia.	(44)

CUMS, chronic unpredictable mild stress; MCAO, middle cerebral artery occlusion; PTZ, pentylenetetrazol; CCI, chronic constrictive injury; LPS, lipopolysaccharide; CRD, colorectal distension; TST, tail suspension test; FST, forced swim test; OFT, open field test; NF-κB, nuclear factor-kappa B; NLRP3, NOD-like receptor family pyrin domain-containing 3; ERK, extracellular signal-regulated kinase; TACE, TNF-α converting enzyme; TNF-α, tumor necrosis factor-α; STAT3, signal transducer and activator of transcription 3; TLR4, toll-like receptor 4; GSDMD, gasdermin D.
